# No systemic reactions to influenza vaccination in egg-sensitized tertiary-care pediatric patients

**DOI:** 10.1186/1710-1492-8-2

**Published:** 2012-03-02

**Authors:** Julia Elizabeth Mainwaring Upton, David Brian Hummel, Anna Kasprzak, Adelle Roberta Atkinson

**Affiliations:** 1 Division of Immunology and Allergy, Hospital for Sick Children, University of Toronto, Toronto, Ontario, Canada

**Keywords:** egg allergy, vaccination, influenza virus, adjuvant, squalene

## Abstract

**Background:**

There are numerous, disparate guidelines for influenza vaccination in egg-allergic patients. We aimed to describe the outcome of selectively applied guidelines, based on risk-stratification, to our high risk, egg-allergic, tertiary-care pediatric population.

**Methods:**

Egg allergy was confirmed with skin testing. The vaccine administered was an adjuvunated 2009 H1N1 influenza A vaccine with < 0.165 mcg/ml ovalbumin. Patients with mild egg allergy were to receive the vaccination in 1 dose, those with severe egg allergy were to receive 2 split doses, and patients with exquisite egg allergy or significant co-morbidities were to be skin tested with the vaccine (prick full strength, intradermal 1:100 of final concentration without adjuvant) and had 5 step desensitization if the testing was positive, or 1-2 step administration if negative. Patients were observed for 60 minutes after the final dose and anaphylaxis treatment was available. We report the frequency of allergic reactions.

**Results:**

Ninety-nine patients were referred and 79 had positive egg testing. Asthma was present in 67% and 30% had prior anaphylaxis to egg. We vaccinated 77 of 79 patients: 71 without performing vaccine skin testing. Two refused vaccination. No patient had a systemic reaction or required treatment. Two patients experienced positive testing to the adjuvanated intradermal vaccine, but were negative without adjuvant.

**Conclusions:**

Our results suggest that most egg-allergic tertiary care pediatric patients can be vaccinated with a low ovalbumin content influenza vaccine without prior vaccine testing. Vaccine skin testing, if used at all, can be reserved for special circumstances. The squalene adjuvant may cause an irritant reaction with intradermal testing.

## Background

Influenza vaccination has traditionally been contraindicated in individuals with egg allergy [1,2] due to the possibility of an allergic reaction to residual egg proteins. Approaches have been recommended to vaccinate egg-allergic individuals. One approach recommends skin testing prior to vaccination [3] and use of a graded challenge [4]. However, there has been evidence demonstrating that the influenza skin testing may not be predictive of reactions [5-7] and intradermal testing has been shown to have an irritant response [8]. There is no such data on intradermal skin testing performance with an adjuvanated influenza vaccine.

Another approach is to give the vaccine in two doses as described in 1997 by James *et al. *[6]. This evidence was used by the 2009 vaccine allergy practice parameters [9] to recommend that if the ovalbumin content is known to be less than 1.2 mcg/ml then the vaccine can be given as 10% followed in 30 minutes by 90%, or as a single dose, without prior testing. In 2009 the Canadian Society of Allergy and Clinical Immunology (CSACI) [10] and a review in the British Medical Journal [11] included a risk-stratification suggestion such that low-risk individuals could be vaccinated in one dose while higher risk would receive split-dosing. However, this approach was not endorsed by some other guidelines, such as the Red Book [1], and the European Academy of Allergy and Clinical Immunology [12].

Adjuvunated influenza vaccines have been used in Europe since 1997 [13]. In the 2009 H1N1 influenza A pandemic (pH1N1) Canada had an adjuvanated influenza vaccine for the first time and it was known that this vaccine had < 0.165 mcg/ml ovalbumin. At the Hospital for Sick Children in Toronto, Canada, we hypothesized that the majority of egg-allergic individuals could be vaccinated with a one or two dose regimen without vaccine prior skin testing, as per the CSACI guidelines, and that vaccine skin testing could be reserved for exquisitely high-risk patients. Importantly, we hypothesized that testing the vaccine itself, without the adjuvant, would reduce the frequency of irritant responses and avoid any potential immunological concerns about intradermal injection of squalene [14]. We demonstrate here the safety and efficacy of our approach in a large group of tertiary care pediatric patients and add to the literature more experience with anaphylactic patients and with the skin testing of squalene-adjuvunated vaccines.

## Methods

The study was conducted at The Hospital for Sick Children, Toronto. The protocol was developed in November 2009. Our inclusion criteria were all patients seen in the Egg Allergy-pH1N1 vaccination clinics. The diagnosis of egg allergy was assessed with commercially available egg extract SPT within the last 6 months. This cohort included children that had never eaten eggs. Patients were excluded if their SPT was negative to eggs, or clearly tolerating eating eggs other than in baked products. Specific IgE levels to egg were not assessed.

**Figure 1:**
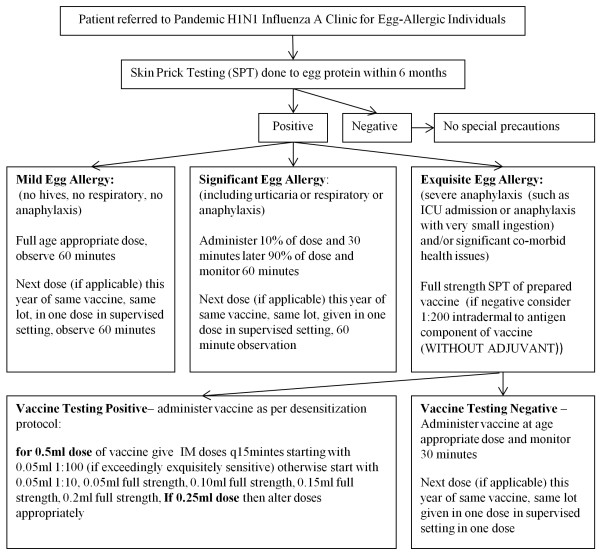
**Protocol for the Management of Egg-Allergic Patients Referred for Adjuvuanted Pandemic H1N1 Influenza A Vaccination**.

Egg-allergic patients were assessed at the time of clinic for the severity of their egg reaction, prior influenza reactions and co-morbid conditions. Figure 1 presents the algorithm provided to physicians. Patients were to be classified as "mild egg allergy" if they had never experienced any generalized reaction of urticaria, angioedema, or respiratory reactions to egg and were to be vaccinated in one dose and be monitored for 60 minutes. This "mild egg allergy" category would include, for example, gastrointestinal reactions alone and patients who had never ingested egg but had positive skin prick tests, Patients were to be classified as "severe egg allergy" if they had any generalized reaction to egg and were to be administered the pH1N1 vaccine in split doses of 10% and then the remainder if there was no reaction. This category of patients would include patients who had urticaria/angioedema to egg, or respiratory reactions to egg, as well as anaphylaxis. The classification of "exquisite egg allergy and/or significant co-morbidity" was to be used if it was determined by the physician that their egg allergy history or their co-morbidities were so severe that skin testing was indicated prior to vaccination. Examples of the type of patient that may be classified in this category may include a patient who experienced anaphylaxis requiring an intensive care admission, or one who experienced anaphylaxis from only touching an egg shell, or a non-verbal patient who would not be able to alert the physician to early signs of an allergic reaction. These patients were to have SPT with full strength vaccine. If negative they were to be intradermally tested with 1:200 of the antigen solution of the vaccine without the adjuvant. This dilution is 1:100 of the final concentration of the vaccine because in vaccine preparation equal parts of antigen vial and adjuvant vial are mixed. If skin tests were positive the vaccine was offered in a 5 step graded challenge. If testing was negative the vaccine was offered in a single or two dose regimen at the physician's discretion.

If the initial vaccine was well tolerated, the follow-up booster vaccine was recommended to come from the same lot as the initial vaccine and could be administered outside this institution. Written consent to vaccination was taken at the time of clinic visit. Patients could refuse vaccination. Anaphylaxis was assessed according to criteria defined by the National Institute of Health in 2006 [15]. The actual decision as to how to manage the patient was guided by protocol but was individualized.

The adjuvunated influenza vaccine used was Glaxo Smith Kline's Arepanrix^®^. It contained less than 0.165 mcg/ml ovalbumin once prepared and typically contained much less (written personal communication, Glaxo Smith Kline). It is an inactivated, split influenza virus vaccine which used the squalene based adjuvant ASO3. The vaccine was administered intramuscularly according to age specific doses. Commercial egg extract, histamine and saline were from Omega Laboratories LTD. A positive test was 3 mm larger than the saline control. SPT was performed with stainless steel lancetters (Medipoint) and intradermal testing was performed with 27 gauge needles.

The main outcome measures were adverse events: minor allergic reaction (hives, angioedema), or serious adverse event (anaphylactic reactions to influenza vaccine). If allergic reactions occurred then a description of the possible predisposing factors was to be attempted. Statistical analysis was descriptive. The occurrence of allergic reactions to the vaccine was to be reported as a simple frequency. This study received approval of the Hospital for Sick Children Research Ethics Board.

## Results

### Patient characteristics

Ninety-nine patients thought to be egg-allergic were referred for administration of the pH1N1 vaccination. Twenty patients were found to be routinely eating egg or had negative skin prick testing to egg and were excluded, thus leaving 79 patients. The patient characteristics are listed in Table 1. A clear history of anaphylaxis to egg was found in 30%, and almost 9% had used epinephrine. One fifth of our patients had reacted to egg within the last 2 years. Most (60%) were avoiding egg entirely and not eating it in baked goods. Over 67% of our patients had asthma and almost 76% had other food allergies. Only 19% of our patients were known to have received the influenza vaccine in the past. All had tolerated the vaccine. Four patients had negative influenza vaccine testing in the past. Almost 4% of our patients had a history of positive influenza testing in prior years and 1% had been desensitized in prior years. On referral, almost 4% of patients had positive testing with the pH1N1 adjuvunated vaccine. Fifteen percent of our patients had other serious medical conditions such as spastic quadriplegia, liver transplants, acute lymphoblastic leukemia, and chromosomal abnormalities.

**Table 1:** Patient Characteristics of The Egg-Sensitized Patients Offered Influenza Vaccine

Variable	**No**.	%	Variable	**No**.	%
**Egg Reactions**			**Other Atopic Disease**		
Anaphylaxis to egg	24	30.4	Asthma	53	67.1
Urticaria and throat tightness	1	1.3	Other Food Allergies	60	75.9
Limited to gastrointestinal system	5	6.3	**Other Significant Co-Morbidities**	79	100.0
Urticaria and/or angioedema	21	26.6	Neurological Conditions		
Pruritis	1	1.3	Significant Hematological Conditions	3	3.8
Mouth sensation	1	1.3	Liver transplants	2	2.5
Perioral rash	3	3.8	T1 DM	3	3.8
Urticaria/angioedema possibly to egg	1	1.3	Significant Failure to Thrive	2	2.5
Unsure of reaction	2	2.5	Trisomy	1	1.3
Positive skin prick test only	17	21.5	**Age at Consultation for H1N1 Vaccine**		
Data not available	3	3.8	0-2 years old		
TOTAL	79	100.0	3-5 years old	12	15.2
Anaphylaxis to unknown food that could be egg	2	2.5	6-9 years old	24	30.4
Recent reaction to egg ( < 2 years)	16	20.3	10-13 years old	28	35.4
**Eating baked egg**			14 years and older	10	12.7
Yes	15	19.0	**Influenza Testing History**	79	100.0
No	48	60.8	+ Test to Influenza Vaccine < 2009	3	3.8
Data not available	16	20.3	+ Test to 2009 H1N1 in Community	3	3.8
**Epinephrine Use**			**Influenza Vaccine History**		
For egg reaction	7	8.9	Received Influenza Vaccine Previously	15	19.0
Unsure if part of treatment for egg reaction	1	1.3	Received Influenza Vaccine by Desensitization	1	1.3
For reaction to unknown food (possibly egg)	3	3.8	Never Had Influenza Vacine	36	45.6
			Unknown	27	34.2
			TOTAL	79	100.0

### Management of patients

**Figure 2:**
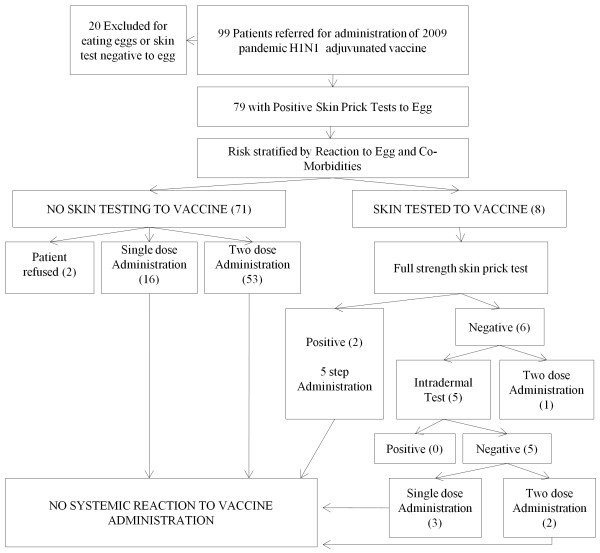
**Flow Diagram Showing Management and Outcome of Patients**.

Figure 2 presents the management of our patients. Of the 79 egg-allergic patients, 71 were not tested to the vaccine and 8 were tested to the vaccine. Of these 71 patients not tested to vaccine, 16 patients were given the vaccine as a single dose, 53 were given the vaccine as a two dose regimen (including 2 with a history of positive influenza vaccine skin test in a prior year), and two patients refused the vaccine (1 decided the wait was too long, 1 did not consent). The 16 patients who were vaccinated in a single dose had the following histories of reaction to egg ingestion: 7 had never eaten egg, 2 had urticaria and/or angioedema, 2 had anaphylaxis (skin and gastrointestinal symptoms), 2 had a perioral rash, 1 had pruritis alone, 1 had urticaria to an unknown food, 1 had gastrointestinal symptoms only, and for one patient there was no data. The 53 patients vaccinated in two doses had the following histories of reaction to egg: 18 had urticaria and/or angioedema, 17 had anaphylaxis to egg, 10 had never eaten egg, 1 had anaphylaxis to an unknown food, 1 had urticaria and throat tightness, 1 had a perioral rash, 2 had gastrointestinal symptoms only, 1 had a reaction to egg not remembered well enough to describe and for 2 patients there was no data. There were 13 patients with "mild egg allergy" based on their histories that were given the vaccine in the more cautious two graded dose regimen whereas the algorithm suggested a single dose. Three patients were given the vaccine in a single dose when two were suggested by our algorithm based on their egg history. No patient had any reaction to the vaccine.

Of the 8 patients that were tested to vaccine, 3 were tested because of positive tests to the pH1N1 adjuvanated vaccine in the community prior to the visit to our clinic by our co-author Dr. Hummel; one had a positive prick test and two had positive testing to the 1:100 intradermal pH1N1 adjuvanated vaccine. The remainder were tested due to histories of severe anaphylaxis. Importantly, the two patients that had positive intradermal tests to the adjuvanated pH1N1 vaccine at 1:100 tested negative without adjuvant. The vaccine was given in two doses to these patients and in one to the other negative testing patients. Two patients had positive prick tests to the vaccine and the administration was performed in a 5 dose regimen. One of these patients had hives at the site of injection on the first diluted dose but no subsequent local or systemic reaction to the subsequent doses. In one patient prick testing was performed due to a fish allergy (the rationale for this testing was squalene adjuvant is fish oil based and the cross reactivity was unknown) and a history of recent anaphylaxis; the testing was negative and a two dose regimen was used. We had no positive intradermal tests to the vaccine without adjuvant. All patients administered the vaccine tolerated it with no systemic adverse event. No patient had any significant reaction to the vaccine.

## Discussion

The egg-allergic patient population we evaluated for vaccination was a tertiary pediatric practice with a high level of anaphylaxis, significant co-morbid conditions, and some with positive flu vaccine tests. Despite this we successfully vaccinated 77 of 79 patients and only skin tested 8/79 patients by using a risk-stratified approach. The only 2 we did not vaccinate were due to patient refusal. No patients had a systemic reaction to vaccination.Some patients received a different administration of vaccine than dictated by their egg allergy alone. There were 13 patients with "mild egg allergy" that were given the vaccine in two graded doses whereas the algorithm suggested a single dose by their egg history alone. Some of these patients had complex medical conditions. For example, one had sickle cell anemia, one had a liver transplant, and one had cerebral palsy and was in a wheelchair. While these conditions are not related to atopy, they may make the recognition and management of a serious allergic reaction more difficult or lessen the parents or physician's comfort level with risk. Administration of vaccine without prior testing was a change from previous years so the use of the perceived more cautious approach (split dosing rather than single dosing) was not surprising. Three patients that were not tested to the vaccine were given the vaccine in a single dose when two were suggested by our algorithm. Two of these patients had hives and angioedema to egg and one had a combined skin and gastrointestinal reaction. These reactions were determined by the treating physician to be mild and were thus triaged to a single dose. None of these patients had a systemic reaction to the vaccine. The algorithm was only a suggestion and the flexibility for the physician or family to choose a more cautious regimen likely helped compliance.

All of our vaccinated patients tolerated the vaccine so it is certainly possible that they would have all tolerated the vaccine in a one dose method, although our patient who received the vaccine in 5 steps had localized hives at the injection site to the first diluted dose; we do not know what the reaction would have been if this patient had received only split dosing or full dosing. Likely this was just a reflection of her cutaneous sensitivity to the vaccine as evidenced by her positive skin tests. A multi-center trial is ongoing to establish the safety of single dosing of influenza vaccine versus split dosing in patients with severe reactions [16] so there may soon be more evidence that even patients with severe reactions can tolerate single dosing without prior vaccine testing.

In addition, there have been a number of papers published on different experiences with the 2009 vaccination campaign in egg-allergic individuals. The largest study to date of influenza vaccination in egg-allergic patients [17] is a prospective study in which patients were risk-stratified by history to receive the vaccine without prior vaccine testing in one dose if low-risk or in a split-dose regimen if they were high-risk. In this study none of 830 vaccinations resulted in anaphylaxis, but one patient needed an antihistamine in the first hour, and one needed salbutamol. Further vaccination of more than 3600 patients resulted in 69 patients developing a possible allergic reaction and two uses of epinephrine. This study supports that egg-allergic patients can receive the egg containing vaccine without prior vaccine testing, but some caution is required.

As of October 2010, the American Academy of Allergy, Asthma and Immunology (AAAAI) no longer recommends routine influenza vaccine skin testing for egg-allergic individuals and has updated guidelines for November 2011 [18] . This organization now recommends a single step vaccine strategy for most patients. A one step or two step approach with no routine skin testing of vaccine is recommended in a recent editorial [19] and in a new focused practice parameter update for the Joint Task Force on Practice Parameters [20]. However, for example, a Cincinnati group [21] and the British Society of Allergy and Clinical Immunology endorse a skin testing based approach [22]. The Red Book [1] has issued an update to their guidelines that some egg-allergic individuals may be vaccinated without prior skin testing with a low albumin vaccine in one or two steps in an appropriate setting, but these recommendations are said to be not applicable to the egg-allergic person with a history of anaphylaxis or severe allergy. Recently, the Advisory Committee on Immunization Practices from the Centers for Disease Control and Prevention issued their recommendations [23] that someone who experiences only hives to eating egg can receive the killed influenza vaccine in one dose with at least a 30 minute wait. Patients with more significant symptoms should be evaluated by an allergist. In October 2011 the Canadian Pediatric Society issued guidelines [24] in which a risk stratification system is recommended to decide between one or two step vaccination with no prior influenza vaccine testing. In these guidelines, people who experienced generalized reactions, including generalized urticaria, to egg would be deemed "higher risk" and vaccinated with a two step regimen. The presence of multiple differing guidelines suggests that more information is still needed.

There are some limitations to our study. Egg allergy was not confirmed by oral challenge, therefore there were likely some skin test positive only children not actually allergic to eggs. However, these children only totaled 17 patients. Another limitation is that only 16 patients (20.3%) had a history of egg reaction within the past 2 years. Therefore, some patients, including some of these patients that we described as having a recent reaction, may have outgrown clinical egg allergy. Follow-up for delayed reactions was by voluntary reporting although our observation period was long enough to assess for most anaphylactic reactions. This vaccine had very low egg content and therefore these findings may not apply to higher egg content vaccines. Our overall patient numbers were small; however our number of patients with a history of anaphylaxis, 24, contributes to the literature because the largest number of patients with a severe reaction receiving an egg containing influenza vaccine reported in a study so far has been the 72/830 reported by Gagnon *et al. *[17].

Risk stratifying patients by their prior reaction to eating egg suggests that the prior history is indicative of their risk of reaction. This presumption may not be true [25]. As another level of caution, in our protocol we suggested that the follow up vaccine (if required) should be from the same lot. This may also be unnecessary [25].

We had two patients referred with positive intradermal testing to the vaccine diluted to 1:100 of the final concentration who did not test positive when tested without adjuvant. Given this potentially irritant response, as well as issues regarding the potential immunological effects of intradermal squalene, we recommend that if squalene containing vaccines need to be intradermally tested they are tested without the adjuvant. Although our numbers are small, to our knowledge there is no other reported experience of intradermal testing of a vaccine with squalene.

## Conclusion

Our study showed that most egg-allergic, tertiary-care pediatric patients can be safely vaccinated with a low-ovalbumin content influenza vaccine without prior vaccine testing and that vaccine testing and desensitization in egg-allergic patients, if used at all, can be reserved for special circumstances. We only vaccine tested patients deemed to have exquisite egg allergy and/or significant co-morbidity (8 of our 79 patients) and only 2 of our patients had a positive prick test and they still tolerated the vaccine in a graded challenge. We found that the adjuvant likely has an irritant response on intradermal testing. This study adds another 24 patients to the current body of evidence that even patients with a prior history of anaphylaxis to egg can receive an egg-containing influenza vaccine. Based on available evidence, many guidelines now conclude that egg-allergic individuals do not benefit from vaccine skin testing prior to low-ovalbumin content influenza vaccination.

## Abbreviations

SPT: skin prick test; CSACI: Canadian Society of Allergy and Clinical Immunology; pH1N1: 2009 H1N1 influenza A pandemic.

## Endnotes

None

## Competing interests

The authors declare that they have no competing interests.

## Authors' contributions

JU contributed to the conception and design of the protocol, the acquisition of and collection of the data, the data entry, the interpretation of the data and drafted the manuscript. DH contributed to the design of the protocol, the collection of the data, and revision of the manuscript. AK contributed to the design of the protocol, the collection of the data and revision of the manuscript. AA contributed to the conception and design of the protocol, the interpretation of the data and revising the manuscript for important intellectual content. All authors read and gave approval to the final manuscript.
